# Erratum to: An improved assembly of the loblolly pine mega-genome using long-read single-molecule sequencing

**DOI:** 10.1093/gigascience/gix072

**Published:** 2017-10-06

**Authors:** Aleksey V. Zimin, Kristian A. Stevens, Marc W. Crepeau, Daniela Puiu, Jill L. Wegrzyn, James A. Yorke, Charles H. Langley, David B. Neale, Steven L. Salzberg

## Abstract

The 22-gigabase genome of loblolly pine (*Pinus taeda*) is one of the largest ever sequenced. The draft assembly published in 2014 was built entirely from short Illumina reads, with lengths ranging from 100 to 250 base pairs (bp). The assembly was quite fragmented, containing over 11 million contigs whose weighted average (N50) size was 8206 bp. To improve this result, we generated approximately 12-fold coverage in long reads using the Single Molecule Real Time sequencing technology developed at Pacific Biosciences. We assembled the long and short reads together using the MaSuRCA mega-reads assembly algorithm, which produced a substantially better assembly, *P. taeda* version 2.0. The new assembly has an N50 contig size of 25 361, more than three times as large as achieved in the original assembly, and an N50 scaffold size of 107 821, 61% larger than the previous assembly.

**Correction**

In the final publication of “An improved assembly of the loblolly pine mega-genome using long-read single-molecule sequencing,” by Aleksey V. Zimin et. al., the article did not include the revised date. The revised date has now been added and the corrected manuscript can be found online at https://academic.oup.com/gigascience/article/6/1/1/2865215/An-improved-assembly-of-the-loblolly-pine-mega?searchresult=1.

The Publisher regrets these errors.

