# Supplementary material for: An improved assembly of the loblolly pine mega-genome using long-read single-molecule sequencing
Source: Gigascience. 2017 Feb 15;6(1):1–4. doi: 10.1093/gigascience/giw016 (PMC5437942; doi:10.1093/gigascience/giw016)
Supplement: GIGA-D-16-00111_Original_Submission.pdf [file giw016_GIGA-D-16-00111_Original_Submission.pdf]

Manuscript
Click here to download Manuscript Loblolly_2.0_assembly_Oct4_submitted_version.docx
Click here to view linked References

An improved assembly of the loblolly pine mega-genome using long-read single-molecule sequencing

Aleksey V. Zimin[1,2], Kristian A. Stevens[3], Marc W. Crepeau[3], Daniela Puiu[2], Jill L. Wegrzyn[4], James A. Yorke[1], Charles H. Langley[3], David B. Neale[5], and Steven L. Salzberg[2,6,*]

[1]Institute for Physical Sciences and Technology, University of Maryland, College Park, MD
[2]Center for Computational Biology, McKusick-Nathans Institute of Genetic Medicine, Johns Hopkins School of Medicine, Baltimore, MD
[3]Department of Evolution and Ecology, University of California at Davis, Davis, CA
[4]Department of Ecology and Evolutionary Biology, University of Connecticut, Storrs, CT
[5]Department of Plant Sciences, University of California at Davis, Davis, CA
[6]Departments of Biomedical Engineering, Computer Science, and Biostatistics, Johns Hopkins University, Baltimore, MD
[*]To whom correspondence should be addressed: salzberg@jhu.edu.

Abstract

The 22 gigabase genome of loblolly pine (*Pinus taeda*) is one of the largest ever sequenced. The draft assembly published in 2014 was built entirely from short Illumina reads, with lengths ranging from 100 to 250 base pairs (bp). The assembly was quite fragmented, containing over 11 million contigs whose weighted average (N50) size was 8,206 bp. To improve this result, we generated approximately 12-fold coverage in long reads using the Single Molecule Real Time (SMRT) sequencing technology developed at Pacific Biosciences. We assembled the long and short reads together using the MaSuRCA mega-reads assembly algorithm, which produced a substantially better assembly, *P. taeda* version 2.0. The new assembly has an N50 contig size of 25,361, more than three times as large as achieved in the original assembly, and an N50 scaffold size of 107,821, 61% larger than the previous assembly.

Introduction

The genome of loblolly pine, first published in 2014 [1], serves as a reference standard for the genetics of this important conifer species, which has been under continuous breeding for more

than 60 years. Previous association studies in loblolly pine have already revealed much about the genetic basis of phenotypic traits [2] and adaptation to the environment [3]; however, these studies examined loci representing a limited number of candidate genes. A reference genome sequence with greater contiguity increases the power of detection and interpretation of association studies. Improvements in the assembly will link together many contigs and scaffolds, and thereby provide a basis for more complete and accurate gene annotation.

With average read lengths now exceeding the contig lengths of most existing conifer genome assemblies, Single Molecule Real Time (SMRT) sequencing technology from Pacific Biosciences (PacBio) has the potential to significantly improve assembly contiguity. To realize this goal for *P. taeda*, a hybrid assembly method was employed, using both PacBio data and pre-existing Illumina sequencing data obtained for the v1.0 assembly [4]. The result of the hybrid approach, presented here, achieves the highest sequence contiguity of any conifer genome to date.

Results

We generated a total of 27,667,399 PacBio reads whose total length was 267 Gb (**Table 1**). Based on an estimated genome size of 22 Gb, this represents approximately 12X coverage of the genome. Because PacBio reads have a relatively high error rate of ~15%, an assembly using only this data would be expected to have relatively poor quality, unless the coverage were much deeper, typically >50X [5]. Therefore we used a hybrid assembly approach, combining the PacBio data with 68X coverage in Illumina reads that was previously generated [4] and then using the MaSuRCA assembler [6] to produce *mega-reads*, a corrected version of the PacBio

reads with an expected accuracy of >99% from which we could generate contigs (see Methods). To produce scaffolds, we used 3.1 billion paired reads from long DNA fragments, of which 1.4 billion were newly generated for this assembly.

Table 1. Summary of raw data, super-reads, and mega-reads for the *Pinus taeda* 2.0 assembly. Coverage is based on a genome size of 22 Gbp. Illumina reads were generated from DNA fragments of 300-500 bp (second row) and from longer 5-10 Kb fragments (third row). Clone coverage refers to the depth of coverage using the entire fragment from which each pair of reads was sequenced (see Methods).

Data type	Number	Total Length (bp)	Mean read length	Coverage	Clone Coverage
PacBio reads	27,667,399	267,426,106,405	9,665	12X	n/a
Illumina reads	10,563,266,162	1,499,483,795,334	142	68X	96X
Illumina reads from 5-10 Kb fragments	3,152,047,806	475,959,218,706	151	22x	69x
Super-reads	96,369,476	44,307,329,021	460	2X	2X
Mega-reads	27,986,125	103,129,750,091	3,685	4.7X	4.7X

The resulting assembly, Ptaeda v2.0, has a total size of 20.6 Gb and an N50 contig size of 25,361 bp (**Table 2**), a three-fold increase over the previously published assembly, Ptaeda v1.01 (GenBank accession GCA_000404065.2). Ptaeda v2.0 has 2.9 million contigs, in comparison to the 16.5 million contigs in Ptaeda v1.01. A closer examination reveals that the primary reason for this dramatic improvement came through the merging of very small contigs: if we consider only contigs longer than 500 bp (**Table 2**), these were reduced in number by just 3.2%. In contrast, the nearly 14 million contigs shorter than 500 bp in Ptaeda v1.01 were reduced by 97%, to just ~410,000 in Ptaeda v2.0.

Considering only the scaffolds longer than 500 bp, Ptaeda v1.01 has 2,158,326 scaffolds, which Ptaeda v2.0 reduces to 1,496,869. Scaffolding relied on paired reads from longer DNA

fragments, ranging from 5-10 Kbp (**Table 1**), most of which were used in the previous assembly (see Methods). The scaffolding improvements were therefore modest compared to the contig improvements. As with the contigs, though, the very short scaffolds, between 200 and 500 bp in length, were dramatically reduced in number, from >7 million to just 1.7 million (**Table 2**). Most of this improvement is a consequence of long PacBio reads that completely contained these short scaffolds.

Table 2. Comparison of two assemblies of *Pinus taeda*, version 1.01 based on Illumina data only, and version 2.0 using the same Illumina data plus 12X coverage in PacBio reads. Total scaffold span includes the sizes of estimated gaps.

Assembly	Ptaeda 1.01	Ptaeda 2.0
Total size	20,148,103,497 bp	20,613,845,687 bp
Total scaffold span	22,564,679,219 bp	22,104,209,064 bp
N50 contig size	8,206 bp	25,361 bp
Number of contigs	16,461,900	2,855,700
Number of contigs > 500bp	2,527,203	2,445,689
N50 scaffold size	66,920 bp	107,036 bp
Number of scaffolds > 200bp	7,068,375	1,762,655
Number of scaffolds > 500bp	2,158,326	1,496,869

Also worth noting is that Ptaeda v2.0 contains 466 Mbp more total sequence than Ptaeda v1.01 (20.614 Gbp versus 20.148 Gbp). Although 466 Mbp is only a small percentage of the total genome size for *Pinus taeda*, it nonetheless represents a substantial amount of sequence, comparable in size to an entire genome for some plants and animals.

Methods

High molecular weight DNA was extracted from pine needles from the same individual tree used for the original *P. taeda* genome [1] using methods previously described [4]. 25 micrograms of DNA was sheared in a Covaris g-tube and subsequently converted to a sequencing library using

the PacBio SMRTbell template kit 1.0 following the manufacturer's instructions (20 kb template preparation using BluePippin size selection) with a low threshold of 15 Kbp. A total of six libraries were made and each was sequenced until depleted. Sequencing utilized four core centers over a period of 9 months to run 332 SMRT cells on RS II sequencers using the P6C4 chemistry and a 240 minute movie length. This yielded 27,667,399 reads with an average length of 9,665 bp and a total length of 267 Gb.

The haploid Illumina sequence data used for this assembly were generated previously [4] using a single megagametophye (haploid tissue extracted from germinated pine seeds). We used 68X coverage in 100-150bp haploid Illumina reads, approximately 1.5 Terabases total, to generate super-reads, accurate longer reads that allow us to compress the overall data set substantially [7]. The Illumina data yielded 96,369,476 super-reads with an average length of 460 (**Table 1**), or approximately 2X coverage of the genome. To scaffold the contigs, we used an additional 1.65 billion pairs (3.1 billion reads) from longer (diploid) fragment libraries, ranging from 5000–10,000 bp in length. These longer-range paired reads (of which 1.4 billion were new, while 1.7 billion were used in the previous Ptaeda1.0 assembly) represent deep clone coverage and helped to join together contigs separated by repeats. *Clone coverage* refers to the depth of coverage of the genome using the full fragments rather than just the sequenced portions; e.g., if fragments are 10,000 bp long and we sequence 100 bp from each end, then the clone coverage will be 10000/200 = 50 times greater than the sequence coverage.

To produce the mega-reads from the PacBio data, and then assemble the mega-reads into contigs, we used the MaSuRCA assembler [6], which has been updated to handle very long reads. The

next step was construction of *mega-reads*, where we tile each PacBio read with super-reads and then replace the PacBio sequence with the more-accurate super-read sequence. The tiling process does not cover every PacBio read fully due to (a) gaps in the Illumina coverage and (b) erroneous insertions in the PacBio reads, but on average most PacBio reads result in fewer than 2 mega-reads. When a PacBio read was split, we used the mega-reads on either side of the corresponding gap to create a synthetic read pair, which was then used in the scaffolding step. A detailed description of the mega-reads algorithm can be found in Zimin et al. [6]. This phase created 27,986,476 mega-reads with an average length of 3,685 bp, approximately 4.7X coverage of the genome.

Because of the relatively low coverage in mega-reads, the assembler used the super-reads in addition to the mega-reads to build the the final set of contigs. We included linking information from the mega-reads and from the long-fragment paired Illumina reads (Table 1) as input to the SOAPdenovo2 scaffolder (Luo et al, 2012) to create the final set of scaffolds.

Assembling the PacBio and Illumina reads took approximately four months on a single 64-core computer with 1 terabyte of RAM. Seven weeks of the total were spent on mega-reads construction and the remaining steps took another 8 weeks.

Availability of data. The Ptaeda 2.0 assembly has been deposited at NCBI under BioProject PRJNA174450, at https://www.ncbi.nlm.nih.gov/bioproject/?term=PRJNA174450.

Acknowledgements

This work was supported in part by the U.S. Department of Agriculture's National Institute of Food and Agriculture under grant 2011-67009-30030, and by the National Institutes of Health under grant R01-HG006677. The authors gratefully acknowledge the assistance of the DNA Technologies Core at the Genome Center of the University of California at Davis, the IGM Genomics Center at the University of California at San Diego, the JHMI Deep Sequencing and Microarray Core Facility at Johns Hopkins University, and the Washington State University Molecular Biology and Genomics Core, all of whom provided sequencing services for this project.

References

1. Neale D.B., J.L. Wegrzyn, K.A. Stevens, A.V. Zimin, D. Puiu, M.W. Crepeau, . . . C.H. Langley. Decoding the massive genome of loblolly pine using haploid DNA and novel assembly strategies. Genome Biology, 2014. **15**(3): R59.
2. Eckert A.J., J.L. Wegrzyn, J.D. Liechty, J.M. Lee, W.P. Cumbie, J.M. Davis, . . . D.B. Neale. The evolutionary genetics of the genes underlying phenotypic associations for loblolly pine (Pinus taeda, Pinaceae). Genetics, 2013. **195**(4): 1353-72.
3. Eckert A.J., A.D. Bower, S.C. Gonzalez-Martinez, J.L. Wegrzyn, G. Coop, and D.B. Neale. Back to nature: ecological genomics of loblolly pine (Pinus taeda, Pinaceae). Molecular Ecology, 2010. **19**(17): 3789-805.
4. Zimin A., K.A. Stevens, M.W. Crepeau, A. Holtz-Morris, M. Koriabine, G. Marcais, . . . C.H. Langley. Sequencing and assembly of the 22-gb loblolly pine genome. Genetics, 2014. **196**(3): 875-90.

5. Berlin K., S. Koren, C.S. Chin, J.P. Drake, J.M. Landolin, and A.M. Phillippy. Assembling large genomes with single-molecule sequencing and locality-sensitive hashing. Nature Biotechnology, 2015. **33**(6): 623-30.

6. Zimin A.V., D. Puiu, M. Luo, T. Zhu, S. Koren, J.A. Yorke, . . . S.L. Salzberg. Hybrid assembly of the large and highly repetitive genome of *Aegilops tauschii*, a progenitor of bread wheat, with the mega-reads algorithm. bioRxiv, 2016: dx.doi.org/10.1101/066100.

7. Zimin A.V., G. Marcais, D. Puiu, M. Roberts, S.L. Salzberg, and J.A. Yorke. The MaSuRCA genome assembler. Bioinformatics, 2013. **29**(21): 2669-77.
