# Supplementary material for: An improved assembly of the loblolly pine mega-genome using long-read single-molecule sequencing
Source: Gigascience. 2017 Feb 15;6(1):1–4. doi: 10.1093/gigascience/giw016 (PMC5437942; doi:10.1093/gigascience/giw016)
Supplement: GIGA-D-16-00111_Revision_2.pdf [file giw016_GIGA-D-16-00111_Revision_2.pdf]

Manuscript
Click here to download Manuscript Loblolly_2.0_assembly_revision2_22Nov.docx

An improved assembly of the loblolly pine mega-genome using long-read single-molecule sequencing

Aleksey V. Zimin[1,2], Kristian A. Stevens[3], Marc W. Crepeau[3], Daniela Puiu[2], Jill L. Wegrzyn[4], James A. Yorke[1], Charles H. Langley[3], David B. Neale[5], and Steven L. Salzberg[2,6,*]

[1]Institute for Physical Sciences and Technology, University of Maryland, College Park, MD
[2]Center for Computational Biology, McKusick-Nathans Institute of Genetic Medicine, Johns Hopkins School of Medicine, Baltimore, MD
[3]Department of Evolution and Ecology, University of California at Davis, Davis, CA
[4]Department of Ecology and Evolutionary Biology, University of Connecticut, Storrs, CT
[5]Department of Plant Sciences, University of California at Davis, Davis, CA
[6]Departments of Biomedical Engineering, Computer Science, and Biostatistics, Johns Hopkins University, Baltimore, MD
[*]To whom correspondence should be addressed: salzberg@jhu.edu.

Abstract

The 22 gigabase genome of loblolly pine (*Pinus taeda*) is one of the largest ever sequenced. The draft assembly published in 2014 was built entirely from short Illumina reads, with lengths ranging from 100 to 250 base pairs (bp). The assembly was quite fragmented, containing over 11 million contigs whose weighted average (N50) size was 8,206 bp. To improve this result, we generated approximately 12-fold coverage in long reads using the Single Molecule Real Time (SMRT) sequencing technology developed at Pacific Biosciences. We assembled the long and short reads together using the MaSuRCA mega-reads assembly algorithm, which produced a substantially better assembly, *P. taeda* version 2.0. The new assembly has an N50 contig size of 25,361, more than three times as large as achieved in the original assembly, and an N50 scaffold size of 107,821, 61% larger than the previous assembly.

Introduction

The genome of loblolly pine, first published in 2014 [1], serves as a reference standard for the genetics of this important conifer species, which has been under continuous breeding for more

than 60 years. The reference genome sequences for loblolly pine and two spruce species are now serving to advance molecular breeding and gene resource conservation programs worldwide in conifers [2]. Previous association studies in loblolly pine have already revealed much about the genetic basis of phenotypic traits [3] and adaptation to the environment [4]; however, these studies examined loci representing a limited number of candidate genes. A reference genome sequence with greater contiguity increases the power of detection and interpretation of association studies. Improvements in the assembly will link together many contigs and scaffolds, and thereby provide a basis for more complete and accurate gene annotation.

With average read lengths now exceeding the contig lengths of most existing conifer genome assemblies, Single Molecule Real Time (SMRT) sequencing technology from Pacific Biosciences (PacBio) has the potential to significantly improve assembly contiguity. To realize this goal for *P. taeda*, a hybrid assembly method was employed, using both PacBio data and pre-existing Illumina sequencing data obtained for the v1.0 assembly [5]. The result of the hybrid approach, presented here, achieves much sequence contiguity than the Illumina-only assembly.

Results

We generated a total of 27,667,399 PacBio reads whose total length was 267 Gb (**Table 1**). Based on an estimated genome size of 22 Gb, this represents approximately 12X coverage of the genome. Because PacBio reads have a relatively high error rate of ~15%, an assembly using only this data would be expected to have relatively poor quality, unless the coverage were much deeper, typically >50X [6]. Therefore we used a hybrid assembly approach, combining the PacBio data with 68X coverage in Illumina reads that was previously generated [5] and then

using the MaSuRCA assembler [7] to produce *mega-reads*, a corrected version of the PacBio reads with an expected accuracy of >99% from which we could generate contigs (see Methods). To produce scaffolds, we used 3.1 billion paired reads from long DNA fragments, of which 1.4 billion were newly generated for this assembly.

Table 1. Summary of raw data, super-reads, and mega-reads for the *Pinus taeda* 2.0 assembly. Coverage is based on a genome size of 22 Gbp. Illumina reads were generated from DNA fragments of 300-500 bp (second row) and from longer 5-10 Kb fragments (third row). Clone coverage refers to the depth of coverage using the entire fragment from which each pair of reads was sequenced (see Methods).

Data type	Number	Total Length (bp)	Mean read length	Coverage	Clone Coverage
PacBio reads	27,667,399	267,426,106,405	9,665	12X	n/a
Illumina reads	10,563,266,162	1,499,483,795,334	142	68X	96X
Illumina reads from 5-10 Kb fragments	3,152,047,806	475,959,218,706	151	22X	69X
Super-reads	96,369,476	44,307,329,021	460	2X	n/a
Mega-reads	27,986,125	103,129,750,091	3,685	4.7X	n/a

The resulting assembly, Ptaeda v2.0, has a total size of 20.6 Gb and an N50 contig size of 25,361 bp (**Table 2**), a three-fold increase over the previously published assembly, Ptaeda v1.01 (GenBank accession GCA_000404065.2). Ptaeda v2.0 has 2.9 million contigs, in comparison to the 16.5 million contigs in Ptaeda v1.01. A closer examination reveals that the primary reason for this dramatic improvement came through the merging of very small contigs: if we consider only contigs longer than 500 bp (**Table 2**), these were reduced in number by just 3.2%. In contrast, the nearly 14 million contigs shorter than 500 bp in Ptaeda v1.01 were reduced by 97%, to just ~410,000 in Ptaeda v2.0.

Considering only the scaffolds longer than 500 bp, Ptaeda v1.01 has 2,158,326 scaffolds, which Ptaeda v2.0 reduces to 1,496,869. Scaffolding relied on paired reads from longer DNA fragments, ranging from 5-10 Kbp (**Table 1**), most of which were used in the previous assembly (see Methods). The scaffolding improvements were therefore modest compared to the contig improvements. As with the contigs, though, the very short scaffolds, between 200 and 500 bp in length, were dramatically reduced in number, from >7 million to just 1.7 million (**Table 2**). Most of this improvement is a consequence of long PacBio reads that completely contained these short scaffolds. Based on the results here using 12X coverage in PacBio reads, we would expect substantially greater contiguity could be obtained for the *P. taeda* assembly if this depth of coverage could be increased substantially.

Table 2. Comparison of two assemblies of *Pinus taeda*, version 1.01 based on Illumina data only, and version 2.0 using the same Illumina data plus 12X coverage in PacBio reads. Total scaffold span includes the sizes of estimated gaps.

Assembly	Ptaeda 1.01	Ptaeda 2.0
Total size	20,148,103,497 bp	20,613,845,687 bp
Total scaffold span	22,564,679,219 bp	22,104,209,064 bp
N50 contig size	8,206 bp	25,361 bp
Number of contigs	16,461,900	2,855,700
Number of contigs > 500bp	2,527,203	2,445,689
N50 scaffold size	66,920 bp	107,036 bp
Number of scaffolds > 200bp	7,068,375	1,762,655
Number of scaffolds > 500bp	2,158,326	1,496,869

Also worth noting is that Ptaeda v2.0 contains 466 Mbp more total sequence than Ptaeda v1.01 (20.614 Gbp versus 20.148 Gbp). Although 466 Mbp is only a small percentage of the total genome size for *Pinus taeda*, it nonetheless represents a substantial amount of sequence, comparable in size to an entire genome for some plants and animals.

To compare the contiguity of the old and new assemblies, we aligned them to an independently sequenced and assembled set of fosmids described previously [5]. We selected all contigs at least 20,000 bp long from one of the large fosmid pools, giving us 2,438 contigs with a total length of 71.97 Mbp. We then aligned these contigs to both Ptaeda v1.01 and Ptaeda v2.0. The results are shown in Table 3. As the table shows, the 2.0 assembly covers slightly more of the total length of all the fosmids with a slightly higher overall percent identity. If we restrict our analysis to fosmid contigs that matched with at least 99.5% identity, Ptaeda 2.0 also looks slightly better, matching 1,138 contigs while Ptaeda 1.01 matches 1,112 contigs.

Table 3. Comparison of alignments of 2,438 contigs assembled from fosmids to each of the two *Pinus taeda* assemblies.

Assembly	Total aligned bases	% of contigs covered	% identity
Ptaeda 1.01	70,296,106	97.67	98.79
Ptaeda 2.0	70,469,590	97.91	98.85

As a limited check on how the improved contiguity might affect annotation, we aligned a set of 458 "core" plant genes from the CEGMA set for *Arabidopsis thaliana* [8] to all contigs and scaffolds from both the 1.01 and 2.0 assemblies of *Pinus taeda*, and to the current assemblies of white spruce (*Picea glauca*, PG29 V4 [9]) and Norway spruce (*Picea abies* [10]). We used tblastn [11] to align the genome assemblies, translated in all six reading frames, to the proteins. We then evaluated the length of the longest-matching segment of each protein to any contig in each assembly. For 50 proteins, the best match to a single contig was longer in the Ptaeda 1.01 assembly, while for 63 proteins, the best match was longer in Ptaeda 2.0. The remaining 345 proteins had best matches of identical lengths in both assemblies.

Table 4. Evaluation of alignments of 458 core (CEGMA) proteins from *Arabidopsis thaliana* to the two *Pinus taeda* assemblies and to two other conifer genomes. Entries show how many proteins have at least 90% of their sequence contained in a single contig (column 2) or scaffold (column 3).

Assembly	Proteins aligned to a single contig (%)	Proteins aligned to a single scaffold (%)
P. taeda 1.01	39	53
P. taeda 2.0	40	45
P. abies 1.0	27	36
P. glauca PG29 V4	27	43

105 If we ask instead how many of these proteins aligned for at least 90% of their length to a single
106 contig, 39% and 40% matched the 1.01 and 2.0 assemblies respectively (**Table 4**). Thus the
107 newer assembly slightly increases the likelihood that most of a gene will be contained within a
108 single contig. When examing the protein alignments to scaffolds, P. taeda 1.01 performs better
109 because that assembly included a separate procedure in which independently-assembled
110 transcripts were used to re-scaffold the genome [5], as did the P. glauca V4 assembly process [9].
111 However, fewer proteins are contained within single contigs or scaffolds for the *P. abies* and *P.*
112 *glauca* assemblies than for either *P. taeda* assembly (**Table 4**).

113 Methods

114 High molecular weight DNA was extracted from pine needles from the same individual tree used
115 for the original *P. taeda* genome [1] using methods previously described [5]. 25 micrograms of
116 DNA was sheared in a Covaris g-tube and subsequently converted to a sequencing library using
117 the PacBio SMRTbell template kit 1.0 following the manufacturer's instructions (20 kb template
118 preparation using BluePippin size selection) with a low threshold of 15 Kbp. A total of six
119 libraries were made and each was sequenced until depleted. Sequencing utilized four core
120 centers over a period of 9 months to run 332 SMRT cells on RS II sequencers using the P6C4
121 chemistry and a 240 minute movie length. This yielded 27,667,399 reads with an average length
122 of 9,665 bp and a total length of 267 Gb.

The haploid Illumina sequence data used for this assembly were generated previously [5] using a single megagametophye (haploid tissue extracted from germinated pine seeds). We used 68X coverage in 100-150bp haploid Illumina reads, approximately 1.5 Terabases in ~15 billion reads (**Table 1**), to generate super-reads, which are accurate longer reads that effectively compress the overall data set substantially [12] (**Figure 1**). The Illumina data yielded 96,369,476 super-reads with an average length of 460 (**Table 1**), or approximately 2X coverage of the genome. To scaffold the contigs, we used an additional 1.65 billion pairs (3.1 billion reads) from longer (diploid) fragment libraries, ranging from 5000–10,000 bp in length. These longer-range paired reads (of which 1.4 billion were new, while 1.7 billion were used in the previous Ptaeda1.0 assembly) represent deep clone coverage and helped to join together contigs separated by repeats. *Clone coverage* refers to the depth of coverage of the genome using the full fragments rather than just the sequenced portions; e.g., if fragments are 10,000 bp long and we sequence 100 bp from each end, then the clone coverage will be 10000/200 = 50 times greater than the sequence coverage.

To produce the mega-reads from the PacBio data, and then assemble the mega-reads into contigs, we used the MaSuRCA assembler [7], which has been updated to handle very long reads. The next step was construction of *mega-reads*, where we tile each PacBio read with super-reads and then replace the PacBio sequence with the more-accurate super-read sequence (**Figure 1**). The tiling process does not cover every PacBio read fully due to (a) gaps in the Illumina coverage and (b) erroneous insertions in the PacBio reads, but on average most PacBio reads result in fewer than 2 mega-reads. When a PacBio read was split, we used the mega-reads on either side

of the corresponding gap to create a synthetic read pair, which was used later in the scaffolding step. During scaffolding, we required at least 2 mates before we joined together a pair of mega-reads. Thus a synthetic read pair was used in scaffolding only if it was confirmed by another read pair spanning the same gap. This step should prevent the creation of chimeric scaffolds in cases where a PacBio read is chimeric. When tiling the high-error-rate PacBio reads with super-reads, the algorithm might on rare occasions merge super-reads from two distinct copies of a repetitive sequence, creating a chimeric mega-read. This would happen if a PacBio read had errors that by chance made a portion of the read appear more similar to the "wrong" copy of a repeat. Such a mega-read, however, will not remain in the assembly unless it is confirmed by another mega-read that is chimeric in the same location, which is highly unlikely because of the random nature of the errors in the PacBio reads. A more detailed description of the mega-reads algorithm can be found in Zimin et al. [7]. This phase of assembly created 27,986,476 mega-reads with an average length of 3,685 bp, approximately 4.7X coverage of the genome.

Because of the relatively low coverage in mega-reads, the assembler used the super-reads in addition to the mega-reads to build the the final set of contigs. We included linking information from the mega-reads and from the long-fragment paired Illumina reads (Table 1) as input to the SOAPdenovo2 scaffolder (Luo et al, 2012) to create the final set of scaffolds.

Assembling the PacBio and Illumina reads took approximately four months on a single 64-core computer with 1 terabyte of RAM. Seven weeks of the total were spent on mega-reads construction and the remaining steps took another 8 weeks.

Availability of data. The Ptaeda 2.0 assembly has been deposited at NCBI under BioProject PRJNA174450, and the PacBio reads are under the same project with accession number SRP034079. Data is also available from the *GigaScience* GigaDB repository[13].

Acknowledgements

This work was supported in part by the U.S. Department of Agriculture's National Institute of Food and Agriculture under grant 2011-67009-30030, and by the National Institutes of Health under grant R01-HG006677 and R01-GM083873. The authors gratefully acknowledge the assistance of the DNA Technologies Core at the Genome Center of the University of California at Davis, the IGM Genomics Center at the University of California at San Diego, the JHMI Deep Sequencing and Microarray Core Facility at Johns Hopkins University, and the Washington State University Molecular Biology and Genomics Core, all of whom provided sequencing services for this project.

References

1. Neale D.B., J.L. Wegrzyn, K.A. Stevens, A.V. Zimin, D. Puiu, M.W. Crepeau, . . . C.H. Langley. Decoding the massive genome of loblolly pine using haploid DNA and novel assembly strategies. Genome Biology, 2014. **15**(3): R59.
2. De La Torre A.R., I. Birol, J. Bousquet, P.K. Ingvarsson, S. Jansson, S.J. Jones, . . . J. Bohlmann. Insights into conifer giga-genomes. Plant Physiol, 2014. **166**(4): 1724-32.
3. Eckert A.J., J.L. Wegrzyn, J.D. Liechty, J.M. Lee, W.P. Cumbie, J.M. Davis, . . . D.B. Neale. The evolutionary genetics of the genes underlying phenotypic associations for loblolly pine (Pinus taeda, Pinaceae). Genetics, 2013. **195**(4): 1353-72.
4. Eckert A.J., A.D. Bower, S.C. Gonzalez-Martinez, J.L. Wegrzyn, G. Coop, and D.B. Neale. Back to nature: ecological genomics of loblolly pine (Pinus taeda, Pinaceae). Mol Ecol, 2010. **19**(17): 3789-805.

5. Zimin A., K.A. Stevens, M.W. Crepeau, A. Holtz-Morris, M. Koriabine, G. Marcais, . . . C.H. Langley. Sequencing and assembly of the 22-gb loblolly pine genome. Genetics, 2014. **196**(3): 875-90.

6. Berlin K., S. Koren, C.S. Chin, J.P. Drake, J.M. Landolin, and A.M. Phillippy. Assembling large genomes with single-molecule sequencing and locality-sensitive hashing. Nat Biotechnol, 2015. **33**(6): 623-30.

7. Zimin A.V., D. Puiu, M. Luo, T. Zhu, S. Koren, J.A. Yorke, . . . S.L. Salzberg. Hybrid assembly of the large and highly repetitive genome of *Aegilops tauschii*, a progenitor of bread wheat, with the mega-reads algorithm. bioRxiv, 2016: dx.doi.org/10.1101/066100.

8. Parra G., K. Bradnam, Z. Ning, T. Keane, and I. Korf. Assessing the gene space in draft genomes. Nucleic Acids Res, 2009. **37**(1): 289-97.

9. Warren R.L., C.I. Keeling, M.M. Yuen, A. Raymond, G.A. Taylor, B.P. Vandervalk, . . . J. Bohlmann. Improved white spruce (Picea glauca) genome assemblies and annotation of large gene families of conifer terpenoid and phenolic defense metabolism. Plant J, 2015. **83**(2): 189-212.

10. Nystedt B., N.R. Street, A. Wetterbom, A. Zuccolo, Y.C. Lin, D.G. Scofield, . . . S. Jansson. The Norway spruce genome sequence and conifer genome evolution. Nature, 2013. **497**(7451): 579-84.

11. Altschul S.F., T.L. Madden, A.A. Schaffer, J. Zhang, Z. Zhang, W. Miller, and D.J. Lipman. Gapped BLAST and PSI-BLAST: a new generation of protein database search programs. Nucleic Acids Res, 1997. **25**(17): 3389-402.

12. Zimin A.V., G. Marcais, D. Puiu, M. Roberts, S.L. Salzberg, and J.A. Yorke. The MaSuRCA genome assembler. Bioinformatics, 2013. **29**(21): 2669-77.

13. Zimin, A, V; Stevens, K, A; Crepeau, M, W; Puiu, D; Wegrzyn, J, L; Yorke, J, A; Langley, C, H; Neale, D, B; Salzberg, S, L (2016): Supporting data for "An improved assembly of the loblolly pine mega-genome using long-read single-molecule sequencing" GigaScience Database. http://dx.doi.org/10.5524/100268

Figure Caption

Figure 1. Construction of super-reads and mega-reads from Illumina reads. Illumina reads (top left) were used to build longer super-reads (green lines), which in turn were used to construct a database of all 15-mers in those reads. For *P. taeda*, each super-read replaced an average of ~150 Illumina reads [5]Table 1). PacBio reads (purple lines) and super-reads were then aligned using the 15-mer database. Inconsistent super-reads are shown as kinked lines; these were discarded and the remaining super-reads were merged, using the PacBio reads as templates, to produce mega-reads. The sequence of the mega-reads was thus derived entirely from the low-error-rate super-reads, not from the raw PacBio reads.

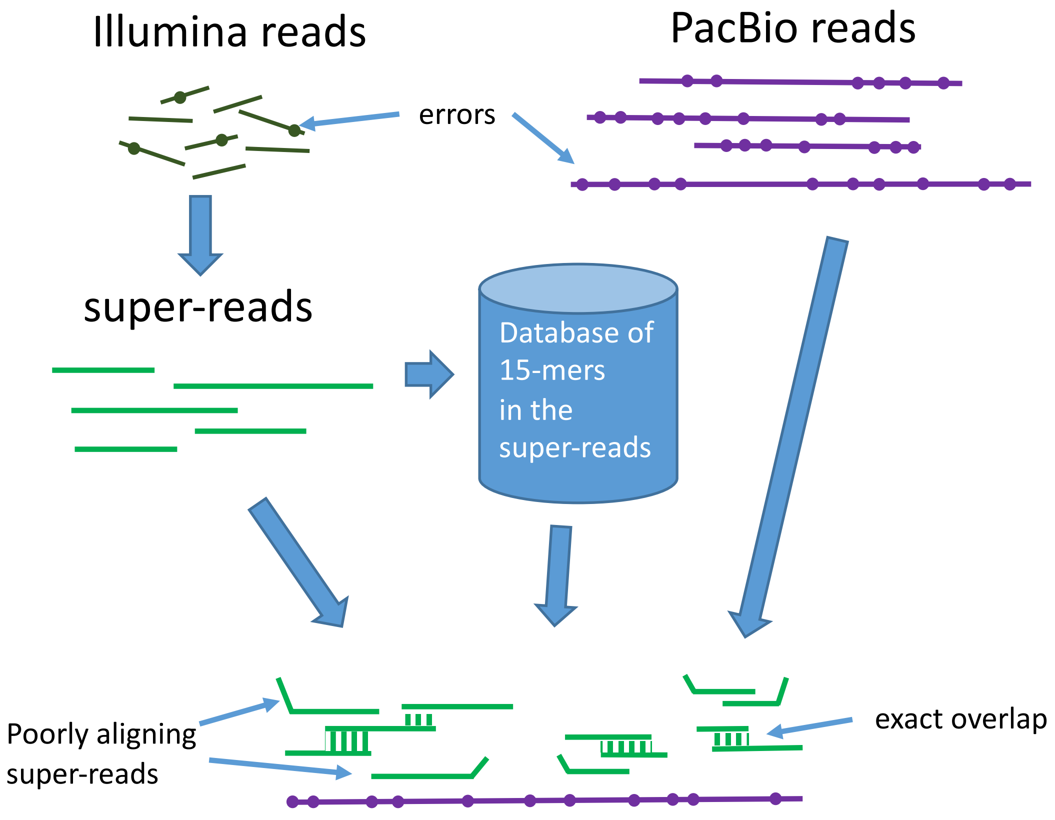
Illumina reads
PacBio reads
errors
super-reads
Database of
15-mers
in the
super-reads
Poorly aligning
super-reads
exact overlap
