# Supplementary material for: An improved assembly of the loblolly pine mega-genome using long-read single-molecule sequencing
Source: Gigascience. 2017 Feb 15;6(1):1–4. doi: 10.1093/gigascience/giw016 (PMC5437942; doi:10.1093/gigascience/giw016)
Supplement: Response_to_Reviewer_Comments_Original_Submission.pdf [file giw016_Response_to_Reviewer_Comments_Original_Submission.pdf]

(Below is the text of our response to reviewers. However, because we included formatted text (italics plus a table), I have uploaded the formatted version as a "personal cover letter" document along with the manuscript.)

Dear Dr. Zauner:
We thank you and the reviewers for your feedback on our manuscript. We have made revisions to address each of the comments from the referees. Below we provide responses for each point, and we describe how we changed our manuscript to address them. We have uploaded the revised manuscript with all of these changes to the Gigascience submission system.
Sincerely,
Steven Salzberg (on behalf of all the authors)

Comments from Editor:

Dear Dr. Salzberg,
Your data note "An improved assembly of the loblolly pine mega-genome using long-read single-molecule sequencing" (GIGA-D-16-00111) has been assessed by two reviewers.

I am happy to say that the reviewers are positive overall and support publication as a Data Note. However, they have raised a number of points which we believe need to be addressed in a revised manuscript.

In particular, both referees would like to see improved evaluation (including BUSCO analysis) of your new assembly, in comparison to the previous one.

We are pleased that the reviewers are positive overall. We provide a point-by-point response explaining our revisions in the text below. For each point, we first quote the reviewer's request or comment, in italic font, and then describe our changes.

Reviewer #1:
Comments:
Could you please report the NG50 length for both contigs and scaffolds (N50 normalized for genome size), using the Assemblathon scripts (using est. 22Gbp genome)? The authors make claims on the "highest sequence contiguity of any conifer genome to date", but do not cite other published work to show that it is indeed the case, nor use metrics that allow for such a comparison. The latest figure reported in a peer-review manuscript for white spruce (P. glauca) scaffolds sits at NG50=114,888 bp (https://gigascience.biomedcentral.com/articles/10.1186/s13742-015-0076-3).

We prefer not to use NG50, which is a recently-introduced statistic that in our view muddies the literature. Prior to the Assemblathon, N50 was consistently used in large numbers of papers to describe assemblies. NG50 introduces an artificial distinction between "true" genome size and the sum of the sizes of the assembled contigs. For most draft genomes, the true size is simply not known, which is problematic when trying to create an NG50. Instead, we consistently state the size N that was used to compute the N50 statistic. This allows assemblies to be compared to one another from one release of a genome to the next, regardless of whether or not the estimated genome size might change.

The reviewer makes a valid point that the P. glauca genome assembly was recently re-scaffolded to generate larger scaffolds, with an N50 of 115 Kb, which is slightly larger than the 108 Kb N50 scaffold size we obtained. We are also in the midst of writing a new paper on the assembly of Douglas-fir, which has an even larger scaffold size. Thus our claim is not accurate and we simply re-worded the sentence to remove it. Rather than "achieves the highest sequence contiguity of any conifer genome to date," the revised text says simply "achieves much higher sequence contiguity than the Illumina-only assembly." (line 47)

The authors mentioned their impetus for seeking a more complete genome with increased assembly contiguity (ie. "basis for more complete and accurate gene annotation"), but do not show how their

improved contiguity results addresses this problem. One assumes that it does, but showing that it indeed does is important.
Whereas the authors are not expected to re-annotate the colossal pine genome assembly for this data note, presenting a summary of core eukaryotic genes (using BUSCO and/or the now defunct CEGMA) in the V1 vs. V2 assembly would substantiate your results and illustrate the importance of assembly improvements, information that would no doubt appeal to a broader readership.

Reviewer 2 also asked if we could look at the core eukaryotic genes. We obtained the BUSCO core gene set for plants by writing to the authors of BUSCO (they only make this set available on request). It turns out the BUSCO software itself is buggy and produced an erroneous report when we ran it on against our assembly, failing to find any genes. Note that we ran it on Drosophila first, and it worked as expected, so we are confident we installed it correctly. (It's possible it simply can't handle such a large genome.) However, the main step is primarily a translated BLAST alignment, so we created translated alignments ourselves to produce a similar type of analysis, which we have added to the paper.

We used the CEGMA core genes from Arabidopsis, which unlike the BUSCO genes are freely available from the CEGMA website (even though the software is no longer supported) at http://korflab.ucdavis.edu/datasets/cegma/. We then aligned these 458 proteins to both the 1.01 and 2.0 assemblies of loblolly, and we found that all proteins were covered nearly completely by both assemblies. However, the main improvement in 2.0 is contig size, so we then looked at a slightly different question: for each protein, what is the length of the longest subsequence that is contained within a single contig? For this question, the longer contigs of assembly 2.0 yield a better result. We added a short new section that explains the results, after line 95:

"As a limited check on how the improved contiguity might affect annotation, we aligned a set of 458 'core' plant genes from the CEGMA set for Arabidopsis thaliana [8] to all contigs from both the 1.01 and 2.0 assemblies of Pinus taeda. We used tblastn [9] to align the genome assembly, translated in all six reading frames, to the proteins. We then evaluated the length of the longest-matching segment of each protein to any contig in each assembly. For 50 proteins, the best match to a single contig was longer in the Ptaeda 1.01 assembly, while for 63 proteins, the best match was longer in Ptaeda 2.0. The remaining 345 proteins had best matches of identical lengths in both assemblies. If we ask instead how many of these proteins aligned for at least 90% of their length to a single contig, 39% and 40% matched the 1.01 and 2.0 assemblies respectively. Thus the newer assembly slightly increases the likelihood that most of a gene will be contained within a single contig."

One point I was not 100% clear on, is the need to pre-process PacBio sequencing data. I think not since the authors discuss the process of read "tiling" on pg 6, line 112, but this point should be clarified in the text. I also wonder whether error-correction (using ECtools, for instance) would help cut down on the assembly run time. Related to the methodology described, the process is difficult to follow due in part by the use of assembler-specific jargon (eg. super-reads, mega-reads). Since you are referring to a non-peer reviewed manuscript for details on the methodology [6], I suggest you add a diagram to this data note, clearly showing the assembly steps involved.

The construction of super-reads is essentially an error-correction step. This was described in a previous publication (ref 10) which is referenced in the methods. The use of super-reads and PacBio reads to create mega-reads is described (as the reviewer notes) in reference 7 which is in bioRxiv and is under review at a journal. We can't use the identical figure without self-plagiarizing, but we created a new, abbreviated version of that figure, showing the two main steps: super-read and mega-read assembly. This diagram is now Figure 1 in the revised paper, which we refer to in the Methods section where we describe the construction of super-reads (line 122) and mega-reads (line 136). The (new) figure caption is as follows: "Figure 1. Construction of super-reads and mega-reads from Illumina reads. Illumina reads (top left) were used to build longer super-reads (green lines), which in turn were used to construct a database of all 15-mers in those reads. For P. taeda, each super-read replaced an average of ~150 Illumina reads (Table 1). PacBio reads (purple lines) and super-reads were then aligned using the 15-mer database. Inconsistent

super-reads are shown as kinked lines; these were discarded and the remaining super-reads were merged, using the PacBio reads as templates, to produce mega-reads. The sequence of the mega-reads was thus derived entirely from the low-error-rate super-reads, not from the raw PacBio reads."

Are the pacbio reads deposited at the SRA? Please provide the accession.

Yes, we have now deposited them in SRA. They are under the same project ID as the assembly, PRJNA174450, and the PacBio reads have accession SRP034079. Under "availability of data" we added a phrase to indicate that both the assembly and the reads are now at NCBI under this project and accession number.

Reviewer #2: An improved assembly of the loblolly pine mega-genome using long-read single-molecule sequencing

[...] Still, as a significant genomic resource, I recommend this paper to be accepted after revision of the below comments

Major comments

* The genome assembly statistics should be complemented with an estimation of the completeness of the gene space. This should include standard tools like CEGMA or BUSCO and be compared both to the Ptaeda 1.01 assembly and other conifer assemblies. It should also include some statistics (eg based on a set of aligned high-quality full-length transcripts) of improved completeness of UTRs or, at the very least, the improvement in assembled sequence length upstream the coding 3' start sites to target potential regulatory regions.

Reviewer 1 had a similar request, which we address in our response above.

* Although the mega-read method has been published elsewhere, the authors should comment on the potential risks for chimeras in tiling super-reads onto high-error PacBio reads in a highly repetitive genome, and clearly justify why their method is applicable in this context.

The tiling of super-reads onto PacBio reads should not introduce chimeras, because we never use the PacBio read only to determine the tiling. The super-reads themselves must overlap significantly before we use the PacBio read as a guide to stitch them together. Wherever the tiling contains a gap, we break the PacBio read apart and tile the left and right parts separately. In the text we explain this as: "the tiling process does not cover every PacBio read fully due to (a) gaps in the Illumina coverage and (b) erroneous insertions in the PacBio reads, but on average most PacBio reads result in fewer than 2 mega-reads." However, the reviewer is correct in that we do create a synthetic read pair that joins the left and right mega-reads in these cases.

These synthetic read pairs are then used in scaffolding. As a check against chimeras, we do not create any scaffolds from synthetic read pairs (i.e., we won't re-join the two halves of a PacBio read that wasn't spanned by super-reads) unless we have at least 2 independent mate pairs that confirm one another. Because chimera are both rare and random, this should make chimera formation very rare; it can only happen if we have two independent PacBio reads that are chimeric in exactly the same place in the genome. We have added the following text after line 150, just after explaining how we create synthetic read pairs, to clarify:
"During scaffolding, we required at least 2 mates before we joined together a pair of mega-reads. Thus a synthetic read pair was used in scaffolding only if it was confirmed by another read pair spanning the same gap. This step should prevent the creation of chimeric scaffolds in cases where a PacBio read is chimeric."

* The fosmid clone resource used for validation in Ref [1] should be used to evaluate the assembly. Are the +20 kbp fosmid contigs used in [1] congruent with the current genome assembly, and if so are they covered in general by longer contigs in v 2.0 than in v 1.01 (as would be expected)? This would serve as an

indication that the improvement in contiguity is real and not an artefact of assembly chimeras.

This is a very good suggestion. To address it, we chose a set of 2,438 assembled fosmids that were 20 Kbp or longer, spanning 72 Mbp, and used them for the evaluation that the reviewer suggests. This represents all the fosmids >20Kb from one of the fosmid pool assemblies described in Ref[1]. These fosmid assemblies are available online on our ftp site: ftp://ftp.ccb.jhu.edu/pub/dpuiu/Pinus_taeda/FosmidPools/LLP-III_E09/SOAPdenovo2.r240/asm-2.contig.20K+
The alignments to these long fosmid contigs show that assembly 2.0 matches the fosmids more closely (though only by a small amount) than 1.01. Here is the newly added paragraph and the new table we have inserted, beginning at line 89, to describe this result:

"To compare the contiguity of the old and new assemblies, we aligned them to an independently sequenced and assembled set of fosmids described previously [5]. We selected all contigs at least 20,000 bp long from one of the large fosmid pools, giving us 2,438 contigs with a total length of 71.97 Mbp. We then aligned these contigs to both Ptaeda v1.01 and Ptaeda v2.0. The results are shown in Table 3. As the table shows, the 2.0 assembly covers slightly more of the total length of all the fosmids with a slightly higher overall percent identity. If we restrict our analysis to fosmid contigs that matched with at least 99.5% identity, Ptaeda 2.0 also looks slightly better, matching 1,138 contigs while Ptaeda 1.01 matches 1,112 contigs."
Table 3. Comparison of alignments of 2,438 contigs assembled from fosmids to each of the two Pinus taeda assemblies.

Assembly	Total aligned bases	% of contigs covered	% identity
Ptaeda 1.01	70,296,106	97.67	98.79
Ptaeda 2.0	70,469,590	97.91	98.85

Minor comments

* In the introduction, it would be fitting with a bit more background on conifer genomics in general with some references to the last years conifer genome projects and what they have brought in terms of new understandings and opportunities. This would put the work into context and guide the reader to better understand the significance of this valuable resource.

We added a sentence to the introduction (lines 33-35), referring to a broader review of this topic: "The reference genome sequences for loblolly pine and two spruce species are now serving to advance molecular breeding and gene resource conservation programs worldwide in conifers [2]." The new reference [2] is: De La Torre AR, Birol I, Bousquet J, Ingvarsson PK, Jansson S, et al. 2014. Insights into conifer giga-genomes. Plant Physiol. 166(4):1–9. We hope this will be sufficient, as we don't think this Data Note is the place for a long discourse on how genome sequencing is benefitting conifer genomes. De La Torre et al discuss this question in their full-length paper.

* Despite a reasonably large effort and a substantial amount of PacBio reads, the final assembly is still very fragmented. It would be of interest if the authors could comment on any predictions they might have based on their gained experience regarding potential further improvements of the assembly with additional PacBio read coverage or other emerging technologies like for example 10X genomics.

To further improve contiguity, we would suggest deeper coverage in PacBio reads before trying any other

strategy. The 12X coverage generated here, although a huge amount of data because of the genome size, is still relatively low coverage compared to other efforts at PacBio-assisted assemblies, which usually generate 30X or more for an Illumina-PacBIo hybrid assembly, and 60X for a PacBio-only assembly. While it would be speculative to discuss this at length in the current manuscript (and it might require much more data to support any assertion), we addressed this comment (which is labelled as minor) by adding the following sentence after line 78, just after we discuss the dramatic reduction in the number of short scaffolds: "Based on the results here using 12X coverage in PacBio reads, we would expect substantially greater contiguity could be obtained for the P. taeda assembly if this depth of coverage could be increased substantially."
