# Supplementary material for: An improved assembly of the loblolly pine mega-genome using long-read single-molecule sequencing
Source: Gigascience. 2017 Feb 15;6(1):1–4. doi: 10.1093/gigascience/giw016 (PMC5437942; doi:10.1093/gigascience/giw016)
Supplement: Response_to_Reviewer_Comments_Revision_1.pdf [file giw016_Response_to_Reviewer_Comments_Revision_1.pdf]

My response includes some formatting as well as a table, so I have included it as a PDF document with the submission. Here I include just the text itself, unformatted and without the table.

We thank you and the reviewers for your feedback on our revised manuscript. We are pleased that reviewer 1 is now satisfied and reviewer 2 only wants clarification of two additional points. We have addressed both of these points and made revisions accordingly, which we explain below. We have uploaded the revised manuscript to the Gigascience submission system.
Sincerely,
Steven Salzberg (on behalf of all the authors)

Comments from Reviewer #2: The manuscript has been revised and improved in accordance with the suggestions. Most importantly, assembly evaluation steps have been included using both core genes and fosmid clone contigs, indicating a slight improvement between v 1.01 and v 2.0. The new Figure 1 is also adding significant value to the manuscript.

There are 2 points made in my initial review that I think should still be addressed before publication:

A) I still suggest a systematic CEGMA comparison to the other conifer genomes, which was not addressed in the revision:
* .. "This should include standard tools like CEGMA or BUSCO and be compared both to the Ptaeda 1.01 assembly and other conifer assemblies."

The authors have evaluated the assembly based on CEGMA core genes in a satisfactory way, but omitted a consistent comparison to other published conifer genome assemblies. This can easily be done and has a value for the readers, so it should be included before publication. In particular, this sentence should be updated to include scores for the P. glauca and P. abies genome assemblies: "..proteins aligned for at least 90% of their length to a single contig, 39% and 40% matched the 1.01 and 2.0 assemblies respectively."

It could also be worth reporting the above metrics both as aligned to "a single contig", but also aligened to ""a single scaffold". So a small table for 90% aligned length CEGMA genes to "single contig", "single scaffold" as columns and "v1.01", "v2.0", "P.glauca", "P.abies" as rows would be very informative in my opinion.

We added this limited CEGMA-like analysis to our study as follows. Note that we don't want to add a lengthy discussion of other conifer genomes, which could distract from our main messages, so we kept this focused on the improvements that longer contigs might yield for annotation. We downloaded the newest P. glauca (PG 29 V4) and P. abies assemblies, and used the same translated alignment method (tblastn) to align the CEMGA genes to these assemblies. (We also added references to both of these genomes.) The results show that only 27% of these genes fell into a single contig in both of the other conifer assemblies (when requiring 90% of the aligned lenth as before), versus 39% and 40% for the two loblolly pine assemblies. This isn't too surprising given that the other conifers have smaller contigs than loblolly pine.

We also looked at scaffolds, where P. taeda 1.01 does the best. This is because our original study added a special, transcriptome-only scaffolding step, where we re-scaffolded the 1.0 assembly using transcripts that were created from RNA-seq data. This produced the 1.01 assembly, in which only a tiny proportion of the scaffolds changed, but all of those scaffolds involved genes. The PG 29 V4 assembly did exactly the same thing: they ran a separate scaffolding step using transcripts, which should significantly improve the number of scaffolds that contain all or most of a gene. Our PacBio assembly does not claim to improve scaffolds, but only contigs, and that's what our paper focuses on. Nonetheless, our 2.0 assembly is better than the other conifers even at the scaffold level (45% versus 36% and 43%), using this CEGMA test, so we included those results in a new table (Table 4) as the reviewer suggested. The original and revised texts are:

Original (lines 98-99): "we aligned a set of 458 'core' plant genes from the CEGMA set for Arabidopsis thaliana [8] to all contigs from both the 1.01 and 2.0 assemblies of Pinus taeda."

Revised: "we aligned a set of 458 "core" plant genes from the CEGMA set for Arabidopsis thaliana [8] to all contigs and scaffolds from both the 1.01 and 2.0 assemblies of Pinus taeda, and to the current assemblies of white spruce (Picea glauca, PG29 V4 [9]) and Norway spruce (Picea abies [10])."

Original (lines 104-106): "If we ask instead how many of these proteins aligned for at least 90% of their length to a single contig, 39% and 40% matched the 1.01 and 2.0 assemblies respectively. Thus the newer P. taeda 2.0 assembly slightly increases the likelihood that most of a gene will be contained within a single contig."
Revised: "If we ask instead how many of these proteins aligned for at least 90% of their length to a single contig, 39% and 40% matched the 1.01 and 2.0 assemblies respectively (Table 4). Thus the newer assembly slightly increases the likelihood that most of a gene will be contained within a single contig. When examing the protein alignments to scaffolds, P. taeda 1.01 performs better because that assembly included a separate procedure in which independently-assembled transcripts were used to re-scaffold the genome [5], as did the P. glauca V4 assembly process [9]. However, fewer proteins are contained within single contigs or scaffolds for the P. abies and P. glauca assemblies than for either P. taeda assembly (Table 4)."

B) I also want to clarify my point on the assembly method, as this comment was not correctly understood by the authors.
* "..the authors should comment on the potential risks for chimeras in tiling super-reads onto high-error PacBio reads in a highly repetitive genome, and clearly justify why their method is applicable in this context."

I apologize for not phrasing this question clearly enough, I was not at all worried about chimeric PacBio reads. I try to explain further:

When aligning super-reads (high-qual) to a PacBio read (low-qual), the alignment must by definition be relatively relaxed. I was therefore worried that a single PacBio read would attract super-reads from different parts of the genome: this would in effect create a chimera (or chimeric scaffold) even if the underlying PacBio read was not. With the huge number of repeats in this genome, the risk of having the same or simliar misalignments re-occuring cannot be neglected, and these created chimeras could potentially make it into the final assembly. Best-case this is rare, and these few cases are filtered out in the assembly process, but worst-case this is common, and many of the final scaffolds are misassemblies. It is extremely hard to evaluate this, since the evaluation metrics used (core genes, fosmid clones) show so little difference between the old and the new assembly (see above), and this uncertainty would be worth commenting on one way or the other. Or, if I have overstated the risk of misassemblies above, it would be good if this could be clarified by the authors to ease my concern.

Thanks for this clarification. Yes, it is possible that the high error rate in PacBio reads could attract super-reads from different parts of the genome, as the reviewer points out. This could create a chimeric mega-read, although such situations are rather unlikely. The risk of misassembly, though, is far lower, because the individual mega-reads have to be assembled together to produce contigs. We don't allow final contigs to consist of just a single unsupported mega-read. So a mis-assembly in the final contig stage will only happen if two independent mega-reads are both chimeric at the same location, which is much less likely (we would argue this is extremely unlikely, though on the scale of a very large genome it might happen).

We left in the text we had added previously, explaining how we would avoid creating chimeric scaffolds from chimeric PacBio reads (at lines 149-150). To address this additional question, we added the following text just after that, in the same paragraph (lines 150 ff.):
"When tiling the high-error-rate PacBio reads with super-reads, the algorithm might on rare occasions merge super-reads from two distinct copies of a repetitive sequence, creating a chimeric mega-read. This would happen if a PacBio read had errors that by chance made a portion of the read appear more similar to the "wrong" copy of a repeat. Such a mega-read, however, will not remain in the assembly unless it is

confirmed by another mega-read that is chimeric in the same location, which is highly unlikely because of the random nature of the errors in the PacBio reads."
