# Supplementary material for: An improved assembly of the loblolly pine mega-genome using long-read single-molecule sequencing
Source: Gigascience. 2017 Feb 15;6(1):1–4. doi: 10.1093/gigascience/giw016 (PMC5437942; doi:10.1093/gigascience/giw016)
Supplement: Reviewer_1_Report_(Original_Submission).pdf [file giw016_Reviewer_1_Report_(Original_Submission).pdf]

Reviewer Report

Title: "An improved assembly of the loblolly pine mega-genome using long-read single-molecule sequencing"

Version: Original Submission **Date:** 10/13/2016

Reviewer name: Rene L Warren

Reviewer Comments to Author:

The authors report on their improved assembly of the loblolly pine genome, using long-read single molecule sequencing from Pacific Biosciences.

The manuscript is concise and well-written, reporting primarily on the contiguity improvements, at the contig and scaffold levels.

I appreciate that sequence assembly of such a large genome is a difficult computational problem and acknowledge that improvements are worthy of mention in the scientific literature.

--Rene

Comments:

Could you please report the NG50 length for both contigs and scaffolds (N50 normalized for genome size), using the Assemblathon scripts (using est. 22Gbp genome)? The authors make claims on the "highest sequence contiguity of any conifer genome to date", but do not cite other published work to show that it is indeed the case, nor use metrics that allow for such a comparison. The latest figure reported in a peer-review manuscript for white spruce (P. glauca) scaffolds sits at NG50=114,888 bp (https://gigascience.biomedcentral.com/articles/10.1186/s13742-015-0076-3).

The authors mentioned their impetus for seeking a more complete genome with increased assembly contiguity (ie. "basis for more complete and accurate gene annotation"), but do not show how their improved contiguity results addresses this problem. One assumes that is does, but showing that it indeed does is important.

Whereas the authors are not expected to re-annotate the colossal pine genome assembly for this data note, presenting a summary of core eukaryotic genes (using BUSCO and/or the now defunct CEGMA) in

the V1 vs. V2 assembly would substantiate your results and illustrate the importance of assembly improvements, information that would no doubt appeal to a broader readership.

One point I was not 100% clear on, is the need to pre-process PacBio sequencing data. I think not since the authors discuss the process of read "tiling" on pg 6, line 112, but this point should be clarified in the text. I also wonder whether error-correction (using ECtools, for instance) would help cut down on the assembly run time. Related to the methodology described, the process is difficult to follow due in part by the use of assembler-specific jargon (eg. super-reads, mega-reads). Since you are referring to a non-peer reviewed manuscript for details on the methodology [6], I suggest you add a diagram to this data note, clearly showing the assembly steps involved.

Are the pacbio reads deposited at the SRA? Please provide the accession

Level of Interest

Please indicate how interesting you found the manuscript: An article whose findings are important to those with closely related research interests

Quality of Written English

Please indicate the quality of language in the manuscript: Acceptable

Declaration of Competing Interests

Please complete a declaration of competing interests, considering the following questions:

- Have you in the past five years received reimbursements, fees, funding, or salary from an organisation that may in any way gain or lose financially from the publication of this manuscript, either now or in the future?
- Do you hold any stocks or shares in an organisation that may in any way gain or lose financially from the publication of this manuscript, either now or in the future?
- Do you hold or are you currently applying for any patents relating to the content of the manuscript?
- Have you received reimbursements, fees, funding, or salary from an organization that holds or has applied for patents relating to the content of the manuscript?
- Do you have any other financial competing interests?
- Do you have any non-financial competing interests in relation to this paper?

If you can answer no to all of the above, write 'I declare that I have no competing interests' below. If your reply is yes to any, please give details below.

I declare that I have no competing interests

I agree to the open peer review policy of the journal. I understand that my name will be included on my report to the authors and, if the manuscript is accepted for publication, my named report including any attachments I upload will be posted on the website along with the authors' responses. I agree for my report to be made available under an Open Access Creative Commons CC-BY license (http://creativecommons.org/licenses/by/4.0/). I understand that any comments which I do not wish to be included in my named report can be included as confidential comments to the editors, which will not be published.

I agree to the open peer review policy of the journal
