# Supplementary material for: An improved assembly of the loblolly pine mega-genome using long-read single-molecule sequencing
Source: Gigascience. 2017 Feb 15;6(1):1–4. doi: 10.1093/gigascience/giw016 (PMC5437942; doi:10.1093/gigascience/giw016)
Supplement: Reviewer_2_Report_(Original_Submission).pdf [file giw016_Reviewer_2_Report_(Original_Submission).pdf]

Reviewer Report

Title: "An improved assembly of the loblolly pine mega-genome using long-read single-molecule sequencing"

Version: Original Submission **Date:** 10/14/2016

Reviewer name: **Bjorn Nystedt**

Reviewer Comments to Author:

An improved assembly of the loblolly pine mega-genome using long-read single-molecule sequencing

This work describes a hybrid assembly approach of the 22 Gbp loblolly pine genome, using 12X PacBio long reads in combination with ~70X Illumina short-reads. The assembly N50 size is reported to improve from 8 kbp to 25 kbp (contigs) and from 67 kbp to 107 kbp (scaffolds) compared to the previously published Illumina-only assembly. The improved assembly was achieved using a previously published version of the MaSuRCA assembler, developed by the same authors.

This improved assembly does not constitute a major leap in the ability to assemble large conifer genomes, but it could still have a significant value for the conifer research community. Longer contiguous sequences (contigs) are important to reconstruct high-quality gene models, which in turn could enable more reliable gene content predictions, improve our understanding of conifer intron/exon structures and UTRs, better resolve issues between real genes and pseudo-genes, and provide an improved resource for evolutionary and functional studies of specific genes. Moreover, improved contiguity, also for the large amounts of short contigs below 1 kbp, could potentially enable improved quantification and evolutionary understanding of the huge amounts of repetitive elements in this genome.

The main weakness of the paper is the lack of evidence for any of the potential down-stream values mentioned above, in particular any improvements in gene annotation. Still, as a significant genomic resource, I recommend this paper to be accepted after revision of the below comments

Major comments

* The genome assembly statistics should be complemented with an estimation of the completeness of the gene space. This should include standard tools like CEGMA or BUSCO and be compared both to the Ptaeda 1.01 assembly and other conifer assemblies. It should also include some statistics (eg based on a set of aligned high-quality full-length transcripts) of improved completeness of UTRs or, at the very least, the improvement in assembled sequence length upstream the coding 3' start sites to target potential regulatory regions.

* Although the mega-read method has been published elsewhere, the authors should comment on the potential risks for chimeras in tiling super-reads onto high-error PacBio reads in a highly repetitive genome, and clearly justify why their method is applicable in this context.

* The fosmid clone resource used for validation in Ref [1] should be used to evaluate the assembly. Are the +20 kbp fosmid contigs used in [1] congruent with the current genome assembly, and if so are they covered in general by longer contigs in v 2.0 than in v 1.01 (as would be expected)? This would serve as an indication that the improvement in contiguity is real and not an artefact of assembly chimeras.

Level of Interest

Please indicate how interesting you found the manuscript: An article of importance in its field

Quality of Written English

Please indicate the quality of language in the manuscript: Acceptable

Declaration of Competing Interests

Please complete a declaration of competing interests, considering the following questions:

- Have you in the past five years received reimbursements, fees, funding, or salary from an organisation that may in any way gain or lose financially from the publication of this manuscript, either now or in the future?
- Do you hold any stocks or shares in an organisation that may in any way gain or lose financially from the publication of this manuscript, either now or in the future?
- Do you hold or are you currently applying for any patents relating to the content of the manuscript?
- Have you received reimbursements, fees, funding, or salary from an organization that holds or has applied for patents relating to the content of the manuscript?
- Do you have any other financial competing interests?

- Do you have any non-financial competing interests in relation to this paper?

If you can answer no to all of the above, write 'I declare that I have no competing interests' below. If your reply is yes to any, please give details below.

I declare that I have no competing interests

I agree to the open peer review policy of the journal. I understand that my name will be included on my report to the authors and, if the manuscript is accepted for publication, my named report including any attachments I upload will be posted on the website along with the authors' responses. I agree for my report to be made available under an Open Access Creative Commons CC-BY license (http://creativecommons.org/licenses/by/4.0/). I understand that any comments which I do not wish to be included in my named report can be included as confidential comments to the editors, which will not be published.

I agree to the open peer review policy of the journal
