# Supplementary material for: An improved assembly of the loblolly pine mega-genome using long-read single-molecule sequencing
Source: Gigascience. 2017 Feb 15;6(1):1–4. doi: 10.1093/gigascience/giw016 (PMC5437942; doi:10.1093/gigascience/giw016)
Supplement: Reviewer_2_Report_(Revision_1).pdf [file giw016_Reviewer_2_Report_(Revision_1).pdf]

Reviewer Report

Title: "An improved assembly of the loblolly pine mega-genome using long-read single-molecule sequencing"

Version: Revision 1 **Date:** 11/18/2016

Reviewer name: Bjorn Nystedt

Reviewer Comments to Author:

The manuscript has been revised and improved in accordance with the suggestions. Most importantly, assembly evaluation steps have been included using both core genes and fosmid clone contigs, indicating a slight improvement between v 1.01 and v 2.0. The new Figure 1 is also adding significant value to the manuscript.

There are 2 points made in my initial review that I think should still be addressed before publication:

A) I still suggest a systematic CEGMA comparison to the other conifer genomes, which was not addressed in the revision:

* .. "This should include standard tools like CEGMA or BUSCO and be compared both to the Ptaeda 1.01 assembly and other conifer assemblies."

The authors have evaluated the assembly based on CEGMA core genes in a satisfactory way, but omitted a consistent comparison to other published conifer genome assemblies. This can easily be done and has a value for the readers, so it should be included before publication. In particular, this sentence should be updated to include scores for the P. glauca and P. abies genome assemblies: "..proteins aligned for at least 90% of their length to a single contig, 39% and 40% matched the 1.01 and 2.0 assemblies respectively."

It could also be worth reporting the above metrics both as aligned to "a single contig", but also aligened to ""a single scaffold". So a small table for 90% aligned length CEGMA genes to "single contig", "single scaffold" as columns and "v1.01", "v2.0", "P.glauca", "P.abies" as rows would be very informative in my opinion.

B) I also want to clarify my point on the assembly method, as this comment was not correctly understood by the authors.

* "..the authors should comment on the potential risks for chimeras in tiling super-reads onto high-error PacBio reads in a highly repetitive genome, and clearly justify why their method is applicable in this context."

I apologize for not phrasing this question clearly enough, I was not at all worried about chimeric PacBio reads. I try to explain further:

When aligning super-reads (high-qual) to a PacBio read (low-qual), the alignment must by definition be relatively relaxed. I was therefore worried that a single PacBio read would attract super-reads from different parts of the genome: this would in effect create a chimera (or chimeric scaffold) even if the underlying PacBio read was not. With the huge number of repeats in this genome, the risk of having the same or simliar misalignments re-occuring cannot be neglected, and these created chimeras could potentially make it into the final assembly. Best-case this is rare, and these few cases are filtered out in the assembly process, but worst-case this is common, and many of the final scaffolds are misassemblies. It is extremely hard to evaluate this, since the evaluation metrics used (core genes, fosmid clones) show so little difference between the old and the new assembly (see above), and this uncertainty would be worth commenting on one way or the other. Or, if I have overstated the risk of misassemblies above, it would be good if this could be clarified by the authors to ease my concern.

Level of Interest

Please indicate how interesting you found the manuscript: An article whose findings are important to those with closely related research interests

Quality of Written English

Please indicate the quality of language in the manuscript: Acceptable

Declaration of Competing Interests

Please complete a declaration of competing interests, considering the following questions:

- Have you in the past five years received reimbursements, fees, funding, or salary from an organisation that may in any way gain or lose financially from the publication of this manuscript, either now or in the future?

- Do you hold any stocks or shares in an organisation that may in any way gain or lose financially from the publication of this manuscript, either now or in the future?
- Do you hold or are you currently applying for any patents relating to the content of the manuscript?
- Have you received reimbursements, fees, funding, or salary from an organization that holds or has applied for patents relating to the content of the manuscript?
- Do you have any other financial competing interests?
- Do you have any non-financial competing interests in relation to this paper?

If you can answer no to all of the above, write 'I declare that I have no competing interests' below. If your reply is yes to any, please give details below.

I declare that I have no competing interests

I agree to the open peer review policy of the journal. I understand that my name will be included on my report to the authors and, if the manuscript is accepted for publication, my named report including any attachments I upload will be posted on the website along with the authors' responses. I agree for my report to be made available under an Open Access Creative Commons CC-BY license (http://creativecommons.org/licenses/by/4.0/). I understand that any comments which I do not wish to be included in my named report can be included as confidential comments to the editors, which will not be published.

I agree to the open peer review policy of the journal
